# Effects of Low-Speed and High-Speed Resistance Training Programs on Frailty Status, Physical Performance, Cognitive Function, and Blood Pressure in Prefrail and Frail Older Adults

**DOI:** 10.3389/fmed.2021.702436

**Published:** 2021-07-26

**Authors:** Hélio José Coelho-Júnior, Marco Carlos Uchida

**Affiliations:** Laboratory of Applied Kinesiology, School of Physical Education, University of Campinas, Campinas, Brazil

**Keywords:** power training, strength training, muscle strength, cognition, elderly

## Abstract

**Aim:** The current study investigated the effects of low-speed resistance training (LSRT) and high-speed resistance training (HSRT) on frailty status, physical performance, cognitive function and blood pressure in pre-frail and frail older people.

**Material and Methods:** Sixty older adults, 32 prefrail and 28 frail, were randomly allocated into LSRT, HSRT, and control group (CG). Before and after intervention periods frailty status, blood pressure, heart rate, and a set of physical performance capabilities and cognitive domains were assessed. Exercise interventions occurred over 16 weeks and included four resistance exercises with 4–8 sets of 4–10 repetitions at moderate intensity.

**Results:** The prevalence of frailty criteria in prefrail and frail older adults were reduced after both LSRT and HSRT. In prefrail, LSRT significantly improved lower-limb muscle strength, while mobility was only improved after HSRT. Muscle power and dual-task performance were significantly increased in both LSRT and HSRT. In frail, LSRT and HSRT similarly improved lower-limb muscle strength and power. However, exclusive improvements in dual-task were observed after LSRT. Memory was significantly increased in prefrail and frail, regardless of the type of resistance training. No significant changes were observed in blood pressure and heart rate.

**Conclusion:** Findings of the present study indicated that both LSRT and HSRT reversed frailty status and improved physical performance in prefrail and frail older adults. Notably, different patterns of improvement were observed among RT protocols. Regarding frailty status, LSRT seemed to be more effective in reverse prefrailty and frailty when compared to HSRT. Greater improvements in muscle strength and power were also observed after LSRT, while HSRT produced superior increases in mobility and dual-task performance. One-leg stand performance was significantly reduced in LSRT, but not HSRT and CG, after 16 weeks. In contrast, RT programs similarly improved verbal memory in prefrail. Finally, no changes in blood pressure and heart rate were observed, regardless of the type of RT.

**Trial Registration:** The protocol was approved by the University of Campinas Human Research Ethics Committee (Protocol No. 20021919.7.0000.5404) and retrospectively registered at ClinicalTrials.gov Protocol Registration and Results System: NCT04868071.

## Introduction

Frailty refers to a reversible state of increased vulnerability to adverse outcomes, including disability and mortality, which occurs separated and faster than the normal aging process in response to a multisystem impairment of the human body and lack of psychosocial support (1–4). Frailty is highly incident in older adults (2, 4) with occurrence rates of 44 new cases per 1,000 person-years (5). In South America, a recent pooled analysis indicated an average prevalence of prefrailty and frailty in community-dwelling older adults of 46.8 and 21.7%, respectively (6). People living in long-term institutions (LTI) are the most affected, so that one-in-two are identified as frail.

With frailty progression, people become more vulnerable to negative events (7–11). Particularly, findings from cross-sectional studies suggested that cognitive function declines across frailty statuses in non-demented older adults (12–14). In addition, frail older people seemed to be at higher risk of dementia in relation to robust individuals (15–17). High blood pressure (BP) levels have also been frequently found in frail people (18–22). A possible explanation for these observations is based on the fact that sustained elevation in arterial BP might predispose to the development of frailty as a result of disturbances in cerebral microcirculation, inflammation and oxidative stress, to quote a few (18–22).

This scenario is especially concerning, since reduced physical performance and declining cognitive function depict the paradigm of unsuccessfully aging (23), while high BP represents a major risk factor for cardiovascular and cerebrovascular diseases (24). As such, frailty represents a major public health problem (25).

The treatment of frailty is under intense debate (26, 27). Among the possible alternatives, considerable attention has been attributed to low-speed resistance training (LSRT), a type of physical exercise in which muscle contractions are performed against a resistance at low-to-moderate velocity (28). Such interest relies in the fact that numerous studies (29–32) have found improvements in frailty-related parameters in older adults who performed LSRT protocols. These findings are reinforced by a recent systematic review (33), which indicated that LSRT might considerably increase lower-limb muscle strength and mobility in frail older adults.

Although these findings are encouraging, just a few of the included studies had identified frailty using a valid scale and investigated exercise programs based on LSRT alone. Moreover, trials have been considered methodologically limited, examined robust people, and have not adopted frailty status as an outcome measure (26, 27). Hence, more studies are still necessary to support the use of LSRT as a first-line therapy to counteract frailty.

Notably, many investigations in the early 2000's started to suggest that muscle power, the capacity to exert force in a short time interval, was more associated with mobility tasks than muscle strength (34–36). These findings led researchers (37–41) to examine whether high-speed resistance training (HSRT), a modality of physical exercise in which muscle contractions are performed as fast as possible (28), could cause greater improvements in mobility tasks than LSRT.

This assumption has been confirmed by numerous investigations conducted with robust (37, 38, 40, 41) and mobility-limited older adults (39), but no studies were performed in frail people. Systematic reviews and metanalyses (42, 43) have supported these results but authors emphasized that data must be carefully extrapolated to the clinical, given that meaningless differences were found among exercise protocols.

Expert opinions (44–48) have encouraged the inclusion of HSRT on exercise programs for frail older adults. According to researchers, perform concentric muscle contractions as fast as possible might be crucial to improve mobility and restore independence. However, empirical evidence comparing the impact of LSRT and HSRT programs on frailty status and related parameters in frail people are scarce (26, 27).

Based on these premises, the current study investigated the effects of LSRT and HSRT on frailty status in pre-frail and frail older people. Secondarily, we examined the effects of both resistance training (RT) programs on physical performance, cognitive function, and BP, given its close association with frailty.

## Materials and Methods

### Study Design

This is a three-arm randomized parallel controlled trial that investigated the effects of two types of RT on frailty status, physical performance, cognitive function, and BP of prefrail and frail older adults. Ethics approval was granted by the University of Campinas Human Research Ethics Committee (Protocol No. 20021919.7.0000.5404) and the study was retrospectively registered at ClinicalTrials.gov (NCT04868071). All participants provided written informed consent prior to participating. All study procedures were conducted following the principles of the Declaration of Helsinki. The present study is in accordance with the CONSORT statement (49).

### Participants

Candidate participants were recruited from two different places, between January 2017 and January 2019. Prefrail volunteers (60–76 years) were recruited from the Senior Center of the city of Poá, SP, Brazil. People were invited to participate by direct contact and through posters placed in the senior center. Volunteers lived alone and were on a waiting list to take part of the exercise programs offered by the senior center. Some of them attended for routine medical appointments.

Frail volunteers (66–99 years) were recruited from a LTI also located in the city of Poá, SP, Brazil. The nursing home is a philanthropic institution structured with accommodations, kitchen, dining and TV rooms, nursing and rehabilitation units, and psychological stimulation room. Most residents arrived at the nursing home due to abandonment, maltreatment, and/or financial, cognitive, and physical disabilities. Patients are accommodated in the rooms according to gender and health status. Residents commonly wake up around 07:00 a.m., are monitored by nurses, and attend to the rehabilitation unit according to their self-will. Physiotherapists offer analgesia, massages, and physical stimulation without overload in individual sessions up to 45 min. In the evenings, older patients watch movies, perform artworks, receive visits, and/or remain in the garden. Visits to theaters, cinemas, parks, and other places occur at least once a month. Meals are offered five times per day and no specific nutritional recommendations (e.g., protein consumption) for older adults are followed.

All candidate participants met the following inclusion criteria: (a) aged 60 years or over; (b) were prefrail or frail according to Fried's criteria (50); (c) performed the sit-to-stand test alone, with a mobility aid, or with the help of a researcher, who provided support but did not interfere in the test performance; (d) possessed sufficient physical and cognitive abilities to understand and perform exercise sessions; and (e) had a physician authorization to participate of physical exercise programs. Exclusion criteria included the clinical diagnosis of orthostatic hypotension, having participated in a structured physical exercise training program in the past 6 months, prescription of hormone replacement therapy and/or psychotropic drugs, and any unstable cardiovascular event (e.g., myocardial infarction) or complication in the past 6 months. Volunteers who had missed four or more exercise sessions in a recurrent and sequential manner according to the records were also excluded.

The power of the sample size was determined using G^*^Power version 3.1.9.2 on the basis of the magnitude of the mean differences among the groups (i.e., for prefrail and after frail). Considering an effect size of 0.75 based on changes in muscle strength (51), a power of 80%, a level of significance set at 5%, and a dropout of 16.9% (52), the sample size necessary was estimated to be of 66 volunteers. Sample size was calculated according to changes in muscle strength, given the lack of studies that used frailty status as a study outcome (26, 27).

A computer-generated list of random numbers was used by an independent researcher to allocate participants into one of three experimental groups using a ratio of 1 1 1 according to age, body mass index (BMI), and sit-to-stand performance: Low-speed resistance training (LSRT), High-speed resistance training (HSRT), and control group (CG), before baseline evaluations.

### Clinical Characteristics

Clinical characteristics were measured at baseline for sample characterization. Body mass and height were measured using an analog weight scale with a Filizola® (Brazil) stadiometer. BMI was calculated according to the following formula:

(a) BMI = body mass (kg)/ height (m^2^);

Information pertaining to disease conditions, medication, schooling, and time of institutionalization was collected through self-report and careful review of medical charts.

### Primary Outcome

#### Frailty Status

The frailty phenotype was adapted from Fried et al. (50) and incorporates measures of multiple physical domains, including weight loss, exhaustion, weakness, slowness, and sedentary behavior (53, 54). Participants were respectively identified as prefrail and frail according to the presence of 1–2 and ≥3 of the following criteria: (1) unintentional weight loss of ≥5 kg in the prior 6 months; (2) self-reported fatigue; (3) weakness, based on isometric handgrip strength (IHG); (4) slowness, based on walking speed (WS); and (5) low physical activity levels according to the short form of the International Physical Activity Questionnaire (IPAQ) (54). Gender-specific and gender- and height-specific cutoff points based on the median values of older adults from Poá, Brazil (55) were used for IHG and WS, respectively. Gender-specific cutoffs were used for physic activity levels (54).

### Secondary Outcomes

#### Physical Performance

Physical performance tests were administered by experienced exercise physiologists and physiotherapists. One examiner was responsible for detailing the operational procedures, showing the test before the assessment, quantifying performance and evaluating motor patterns. The other examiner ensured participants' safety by providing occasional verbal and/or tactile cueing if needed. Particularly, most frail participants needed physical support for performing mobility tests, which was provided by the research team without interfering in the performance. After the explanation and before each test, prefrail participants performed a familiarization trial to ensure they had fully understood each test, while frail participants were requested to verbally explain the tests, to avoid fatigue. Except for the 6-min walking test (6MWT), participants performed all tests twice with the mean result used for analysis. Tests were administered in a sequential order with a 2–10-min rest interval, as follows: (1) IHG (56), (2) muscle strength of knee extensors, hip flexors, and ankle extensors (57); (3) one-leg stand (58); (4) balance tests of the Short Physical Performance Battery (SPPB) (59); (5) sit-to-stand (59); (6) Timed “Up and Go” (TUG) (60); (7) WS at usual and fast paces (61); and (8) 6MWT (59). A detailed description of physical performance tests and test reliability values are available in Supplementary Material 1.

## Cognitive Function

Cognitive tests were administered face-to-face in a private silent room by a trained researcher. Global cognitive function was assessed using the mini-mental state examination (MMSE) (62, 63) and the clock drawing test (CDT) (64). Attention, inhibitory control, and reaction time (ms) were assessed using a computerized version of the Stroop test (TESTINPACSTM) (65, 66). The Rey's auditory verbal learning test (RAVLT) (67–70) was used to assess episodic and delayed memory, and susceptibility to interference. The test consists of read-aloud two lists (A and B) of 15 substantives each (with a 1-s interval between each word). At the beginning of the test, list A was read five consecutive times by a researcher. Then, participants were requested to recall as many words were possible after each trial (A1-A5). The list B, interference list, with new 15 substantives was read after A5 and words were retrieved (B1). Finally, participants were asked to recall the words from list A immediately after the interference list (A6, immediate recall) and after a delay of 20 min (A7, delayed recall), without listening to the list A again.

Four summary scores were calculated (71), as follows:

(b) Verbal learning (VL) score = ∑A1-A5–(5 ^*^ A1);
(c) Proactive interference (PI) = B1/A1;
(d) Retroactive interference (RI) = A6/A5;
(e) Forgetting speed (FS) = A7-A6;

Final scores are provided as continuous data and no specific cutoff points were used.

A detailed description of cognitive tests is available in Supplementary Material 1.

### Blood Pressure and Heart Rate

BP was measured accordingly to the VII Joint National Committee of High Blood Pressure (JNC7) (72). Pre- and post-intervention BP values were based on the mean values measured in three consecutive visits in three different days. For BP evaluation, participants remained seated in a comfortable chair in a room with artificial light. BP and heart rate (HR) were blindly measured in the left arm using automated oscillometric equipment (BP 3BT0A, Microlife AG, Widnau, Switzerland) (73). At the end of each measurement, the equipment provided systolic BP (SBP), diastolic BP (DBP), and HR.

### Exercise Interventions

Exercise interventions were carried out over a total of 16 weeks in the mornings (08:00 a.m.−12:00 a.m.) under the supervision of fitness instructors and physiotherapists. Exercise sessions for frail participants were performed individually and occurred in the LTI, while prefrail people attended to the senior center and performed exercise sessions in groups of 3–4 older adults. The first 4 weeks were dedicated to participants' familiarization. In this period, four exercises for lower limbs: (1st) squat on the chair, (2nd) seated unilateral hip flexion, (3rd) seated unilateral knee extension, and (4th) bilateral calf raise with 12–15 submaximal repetitions avoiding fatigue (i.e., inability to complete a repetition in a full range of motion) were performed. The number of sets was increased linearly during the first month, so that one set was performed in the 1st week, two sets in the 2nd week, 3 sets in the 3rd week, and 4 sets in the 4th week. The main exercise period occurred in the consecutive 12 weeks. After a brief warm-up, participants performed the same four exercises utilized during the familiarization period using adjustable weight vests and ankle weights (DOMYOS®, Shanghai, China). The total volume (sets × repetitions × load) was equalized among the groups. However, LSRT and HSRT were designed according to the peculiarities of each type of RT (28, 74). Hence, the LSRT group performed four sets of 8–10 repetitions at 70–75% of 1-repetition maximum (1RM). The concentric and eccentric phases were carried out for ~2.5-s. For HSRT, exercises were performed 8 times (sets) with 3–5 repetitions at 70–75% of 1RM. The concentric phase was performed as fast as possible, and the eccentric phase was carried out for ~2.5-s. No maximal strength test was conducted to determine the load of bilateral calf raise, so that participants performed this exercise using the same load that was used to seated unilateral knee extension exercise. A researcher was responsible for monitoring and ensuring that the velocity of muscle contractions was adequate to the protocol. Verbal encouragement was provided to HSRT.

### Ten-Repetition Maximum Test (10RM)

10RM tests were performed prior, monthly, and at the end of the exercise programs in the following three exercises: squat on the chair (until 90° knee flexion), seated unilateral hip flexion, and seated unilateral knee extension. Before the tests, individuals performed a brief specific warm-up using light loads. Afterwards, the 10RM load was determined up to five attempts, with a 3-min interval between the attempts. The resistance was increased according to the capacity of the volunteer to perform more than one successful repetition maximum with the proper technique. The test was completed when participants were unable to perform more than 10 repetitions using a proper technique (75). All trials were performed with participants using the full range of motion. Subsequently, the 1RM was calculated based on the following formula:

(f) 1RM = (10RM/(1.0278−[0.0278 × 10])) (76).

### Control Group

The CG performed flexibility sessions for 20 min once a week.

### Statistical Analysis

Normality of data was ascertained using the Kolmogorov-Smirnov test. Data are presented as mean ± standard deviation (SD) or absolute numbers (percentages) for continuous and categorical variables, respectively. A group × time repeated-measures ANOVA followed by Bonferroni *post hoc* analyses were performed to determine whether there were significant differences between groups. For all tests, the level of significance was set at 5% (*p* < 0.05). All analyses were conducted using GraphPad Prism 6.0. (San Diego, CA).

## Results

**Figure 1:**
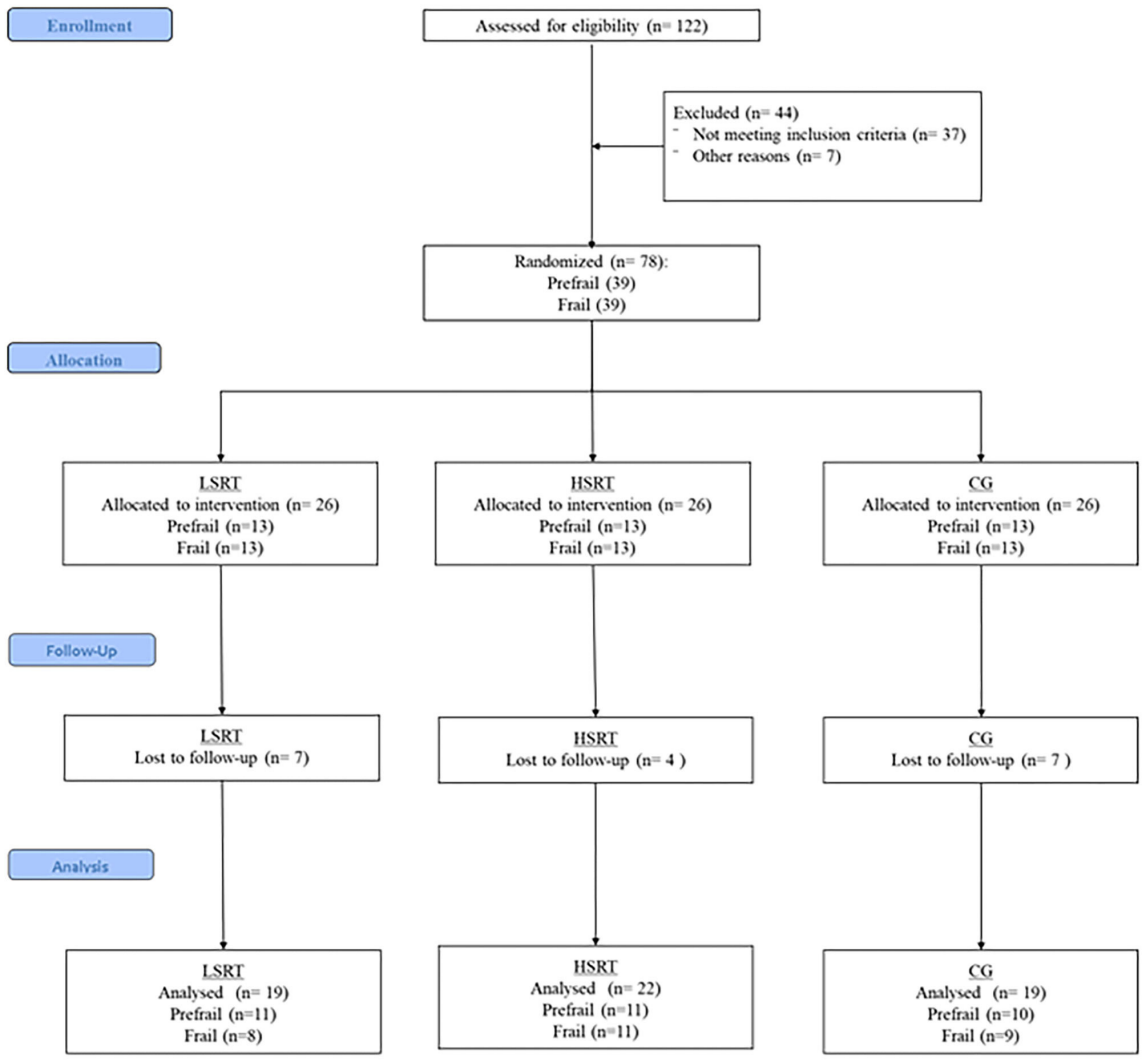
Flowchart of the present study. LSRT, Low-speed resistance training; HSRT, High-speed resistance training; CG, Control group.

One-hundred twenty-two older adults were recruited and evaluated according to the eligibility criteria. Of these, 37 were identified as robust and seven could not attend exercise training in the mornings, leaving a total of 78 older adults, 39 prefrail and 39 frails, who were randomized into the three groups (i.e., LSRT, HSRT, and CG). Adherence to exercise sessions was above 95% in both prefrail and frail groups. Five prefrail and 11 frail participants withdrew from the trial. In prefrail, three participants from the CG withdrew to start a programmed exercise program, while two, one from the HSRT and one from the LSRT, withdrew after 2 weeks because they were not randomized to the same exercise group. In frail, four participants withdrew due to personal reasons, two participants due to the 10RM test, one start to take psychotropic drugs, one could not attend for exercise sessions for 2 months due to substantial weight loss and complains of muscle fatigue, one had a stroke, one had urinary tract infection, and one died. The flowchart of the present study is shown in Figure 1.

Most frail participants complained of extraneous muscle fatigue during the familiarization period, but not in the main period. Two participants reported joint pain and one frail participant from the HSRT group reported epigastric discomfort and nausea during the performance of the squat on the chair exercise. No falls were recorded in pre-frail community dwelling-older adults during the protocol. In frail, six falls (four in the same participant) were registered in the HSRT, four in LSRT, and four in the CG. All falls occurred on days other than training days.

### Clinical Characteristics

Table 1 shows the clinical characteristics of prefrail and frail participants according to group allocation. There were no significant differences in clinical characteristics between experimental and CG groups, regardless of frailty status. Frail participants were older and had less formal education in comparison to prefrail. The average BMI was within normal limits for both groups. Hypertension and type II diabetes were highly prevalent in prefrail and frail, while osteoarthritis, stroke, and Parkinson's disease were most notorious in frail. There were significant differences in physical performance between exercise and CG in prefrail and frail. In prefrail, LSRT showed higher right and left muscle strength of knee extensors, right hip flexor, and balance on one-leg stand test. In addition, CG showed higher TUG performance when compared to LSRT. In frail, LSRT showed higher right and left muscle strength of knee extensors in comparison with HSRT and CG, and lower TUG performance in comparison to HSRT. No differences in cognitive function or BP were observed in any group.

**Table 1:** Main characteristics of study participants.

	**Prefrail (*****n =*** **32)**				**Frail (*****n =*** **28)**			
	**LSRT (*n =* 11)**	**HSRT (*n =* 11)**	**CG (*n =* 10)**	**Total (*n =* 32)**	**LSRT (*n =* 8)**	**HSRT (*n =* 11)**	**CG (*n =* 9)**	**Total (*n =* 28)**
**Variables**								
**Clinical characteristics**								
Age, years	65 ± 3.5	65 ± 2.8	65 ± 3.5	65 ± 3.2	75 ± 4.6	73 ± 7.5	75.0 ± 9.2	76 ± 7.2
Gender, female/male	9/2	11/0	11/0	31/2	6/2	6/5	6/3	18/10
BMI, kg/m^2^	26.8 ± 5.7	24.5 ± 2.4	25.5 ± 3.3	26.3 ± 4.5	25.3 ± 3.1	24.6 ± 3.5	25.7 ± 2.4	24.8 ± 5.3
Schooling, years	7 ± 2.9	4 ± 2.1	8 ± 2.1	6 ± 2.8	2 ± 4.5	0 ± 1.0	1.8 ± 2.4	1.5 ± 2.7
Time of institutionalization, years	—	—	—	—	2 ± 0.9	2 ± 3.1	2 ± 1.5	2 ± 2.2
**Comorbidities, %**								
Hypertension	72.7	36.6	100	78.7	87.5	63.6	44.4	64.3
Osteoarthritis	27.2	27.2	36.3	34.3	25.0	36.3	66.6	45.5
Stroke	0	0	0	0	12.5	9.0	11.1	10.7
Diabetes	9.0	27.2	9.0	17.4	37.5	9.0	11.1	17.8
Parkinson's disease	0	0	0	0	0	9.0	0	3.5
**Frailty phenotype, %**								
Weakness	45.4	72.7	0	40.6	87.5	72.7	77.7	78.5
Slow walking speed	18.1	45.4	20.0	28.1	87.5	81.8	66.6	78.5
Unintentional weight loss	0	9.0	40.0	15.6	50	63.6	77.7	64.2
Exhaustion	45.4	72.7	81.8	66.2	100	100	100	100
Low activity level	0	9.0	20.0	9.3	100	100	100	100
**Physical performance**								
Right IHG, kg	25.0 ± 4.0	21.9 ± 5.7	25.9 ± 3.2	24.2 ± 4.7	6.2 ± 5.5	4.8 ± 6.4	13.8 ± 13.7^a^^,^^b^	8.1 ± 9.8
Left IHG, kg	25.5 ± 6.1	21.3 ± 6.0	25.7 ± 3.6	24.2 ± 5.7	8.5 ± 9.5	9.6 ± 9.3	12.7 ± 12.4^a^^,^^b^	10.3 ± 10.2
Right knee extensor, kgf	17.3 ± 4.2	11.7 ± 2.3^a^	10.1 ±1.9^a^	13.4 ± 4.4	7.0 ± 1.9	7.1 ± 2.8	7.0 ± 5.7	6.6 ± 4.1
Left knee extensor, kgf	14.8 ± 3.1	12.3 ± 3.4^a^	10.3 ± 2.3^a^	12.7 ± 3.5	6.6 ± 2.0	6.1 ± 3.7	6.6 ± 5.0	6.2 ± 3.9
Right hip flexor, kgf	11.1 ± 3.2	8.2 ± 3.3^a^	8.6 ± 3.6^a^	9.4 ± 3.5	6.0 ± 1.7	5.4 ± 2.2	4.7 ± 2.8	5.0 ± 2.6
Left hip flexor, kgf	10.1 ± 2.7	8.1 ± 2.8	8.3 ± 2.5	8.9 ± 2.8	5.4 ± 1.1	5.1 ± 2.5	4.3 ± 2.5	4.8 ± 2.3
Right ankle extensor, kgf	6.8 ± 2.1	6.4 ± 1.8	5.8 ± 1.1	6.4 ± 1.8	5.6 ± 1.5	4.3 ± 2.6	3.8 ± 2.3	4.2 ± 2.5
Left ankle extensor, kgf	7.1 ± 1.7	6.4 ± 1.8	6.4 ± 1.1	6.7 ± 1.6	3.8 ± 2.8	4.4 ± 2.4	3.7 ± 2.6	3.9 ± 2.6
Right one-leg stand, s (30 s max)	19.4 ± 9.7	10.9 ± 11.6^a^	12.5 ± 12.0^a^	14.4 ± 11.4	0.1 ± 0.3	0.1 ± 0.4	2.2 ± 3.1	0.8 ± 2.0
Left one-leg stand, s (30 s max)	16.4 ± 11.0	13.0 ± 12.2	7.3 ± 10.4^a^	12.4 ± 11.6	0.0 ± 0.2	0.2 ± 0.4	2.3 ± 4.4	0.9 ± 2.6
Normal balance, s (10 s max)	10.0 ± 0.0	9.8 ± 0.6	10.0 ± 0.0	9.9 ± 0.4	1.2 ± 3.5	1.8 ± 4.0	4.4 ± 5.2	2.5 ± 4.4
Semi tandem balance, s (10 s max)	10.0 ± 0.0	9.8 ± 0.6	10.0 ± 0.0	9.9 ± 0.4	0.0 ± 0.0	1.0 ± 3.0	4.4 ± 5.2	1.9 ± 3.9
Tandem balance, s (10 s max)	10.0 ± 0.0	6.9 ± 0.6	10.0 ± 0.0	8.9 ± 3.1	0.0 ± 0.0	0.8 ± 2.7	5.5 ± 5.2	0.7 ± 2.5
Sit-to-stand, s	8.4 ± 1.1	10.0 ± 2.3	8.0 ± 0.6	8.9 ± 2.0	26.7 ± 11.6	26.2 ± 13.3	28.6 ± 10.9	25.3 ± 10.4
TUG at usual pace, s	8.0 ± 0.8	10.2 ± 2.7	6.2 ± 1.4^a^	8.3 ± 2.5	119.8 ± 180.2	20.8 ± 27.3^a^	46.4 ± 36.3	57.3 ± 104.0
TUG at fast pace, s	6.5 ± 1.1	8.4 ± 2.5	5.6 ± 0.9	6.9 ± 2.0	38.0 ± 46.3	17.4 ± 22.8^a^	28.5 ± 25.4	26.9 ± 31.9
TUG with verbal task, s	8.3 ± 1.0	10.7 ± 3.9	7.1 ± 1.2	8.8 ± 2.9	69.0 ± 109.8	18.4 ± 24.1	37.5 ± 43.2	36.6 ± 62.5
TUG with motor task, s	8.7 ± 1.7	10.1 ± 2.1	8.0 ± 0.8	9.0 ± 1.9	14.2 ± 13.0	7.1 ± 12.9	16.1 ± 20.7	11.5 ± 15.4
TUG with both verbal and motor tasks, s	8.3 ± 1.1	11.6 ± 3.2	10.9 ± 1.4	10.3 ± 2.5	17.6 ± 19.7	8.3 ± 18.7	17.7 ± 23.2	12.8 ± 19.8
WS at usual pace, m/s	1.3 ± 0.3	1.2 ± 0.2	1.3 ± 0.3	1.3 ± 0.3	0.41 ± 0.37	0.81 ± 0.99	0.51 ± 0.41	0.50 ± 0.35
WS at fast pace, m/s	1.8 ± 0.3	1.5 ± 0.3	1.9 ± 0.3	1.8 ± 0.4	0.46 ± 0.41	0.66 ± 0.91	0.62 ± 0.50	0.57 ± 0.40
6MWT, m	480 ± 137	460 ± 151	589 ± 179	507.7 ± 161.2	150 ± 174	100 ± 136	91.4 ± 107	78.1 ± 118.4
**Cognitive function**
MMSE, points	24.3 ± 1.9	23.2 ± 1.8	23.4 ± 1.5	24.4 ± 2.4	15.6 ± 4.5	13.8 ± 3.7	16.0 ± 2.0	14.9 ± 3.6
CDT, points	1.6 ± 0.8	1.6 ± 0.7	2.0 ± 0.7	1.7 ± 0.6	5.5 ± 1.4	5.5 ± 1.3	4.4 ± 1.4	5.2 ± 1.3
**Hemodynamic parameters**
SBP, mmHg	130.4 ± 14.9	131.6 ±19.5	137.8 ± 13.5	133.0 ± 16.1	124.0 ± 21.6	114.3 ± 17.0	140.1 ± 15.4	124.8 ± 20.5
DBP, mmHg	68.0 ± 23.0	72.0 ± 10.0	79.8 ± 11.8	73.1 ± 16.4	81.9 ± 15.5	67.8 ± 9.4	79.2 ± 11.7	76.4 ± 13.8
HR, bpm	73.7 ± 11.6	73.7 ± 9.6	73.1 ± 4.2	73.6 ± 8.8	65.9 ± 5.6	70.5 ± 12.2	86.7 ± 12.3	80.2 ± 12.9

*BMI, Body mass index; IHG, isometric handgrip strength; TUG, Timed “Up-and-Go”; 6MWT, 6-min walking test; MMSE, Mini mental state examination; CDT, Clock Drawing Test; SPB, Systolic Blood Pressure; DBP, Diastolic Blood Pressure; HR, Heart Rate; LSRT, Low-Speed Resistance Training; HSRT, High-Speed Resistance Training; CG, Control Group*;

a *P < 0.05 vs. LSRT*;

b *P < 0.05 vs. HSRT*.

### Frailty Status

**Figure 2:**
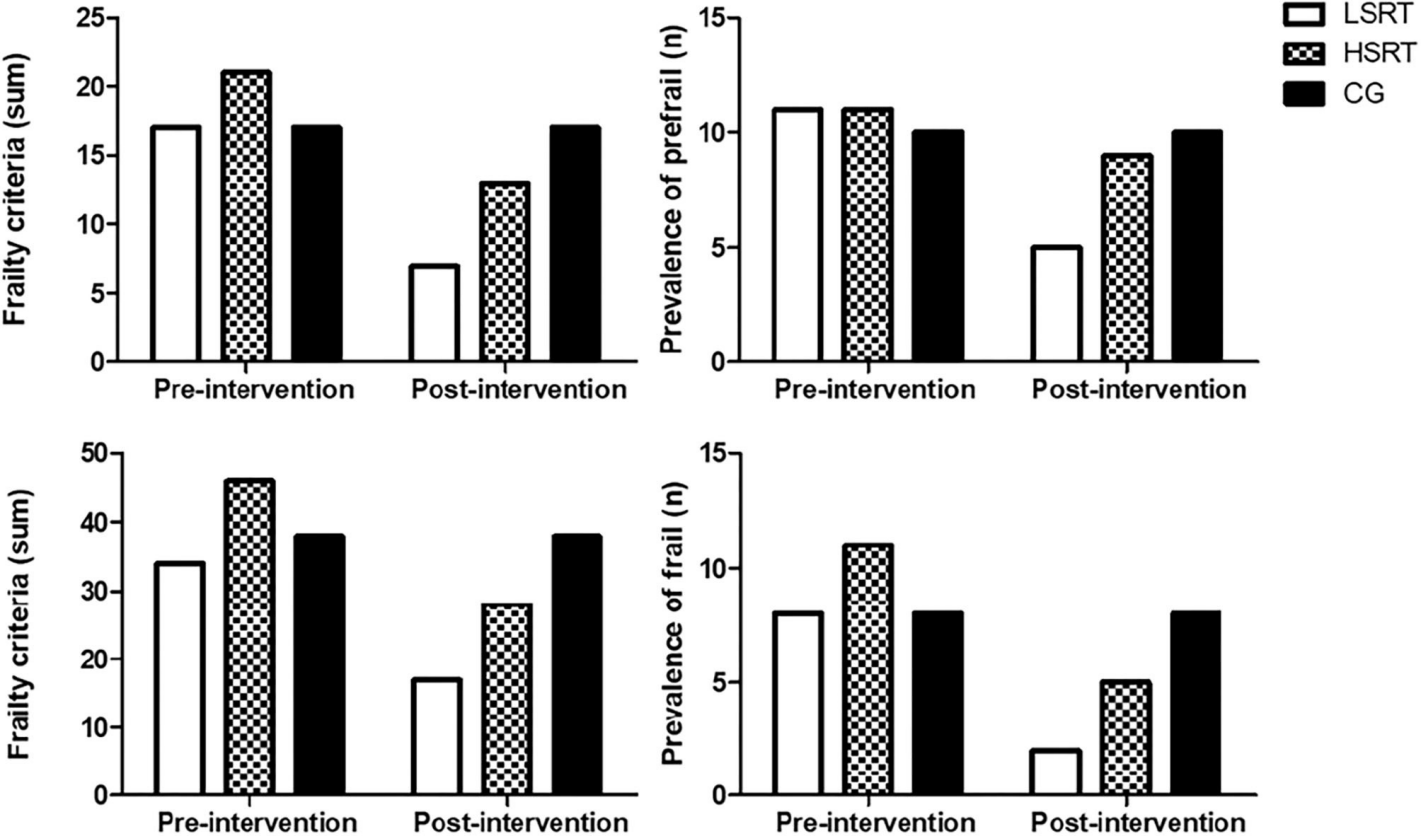
Effects of RT on Frailty Status in prefrail and frail older adults. LSRT, Low-speed resistance training; HSRT, High-speed resistance training; CG, Control group.

The effects of RT on frailty status are shown on Figure 2. Both LSRT and HSRT reduced the prevalence of frailty criteria in prefrail and frail older adults. Six (54.5%) prefrail participants returned to robust condition after LSRT, while two (18.1%) participants became robust after HSRT. RT improved weakness (LSRT, *n* = 1; HSRT, *n* = 0), slowness (LSRT, *n* = 2; HSRT, *n* = 1), and exhaustion (LSRT, *n* = 8; HSRT, *n* = 6) in prefrail. In frail, 10 participants, five in each intervention group (62.5%, 45.4%), returned to prefrail condition, and two participants (12.5%, 9.0%), one in each intervention group, returned to robust condition after LSRT and HSRT, respectively. RT improved weight loss (LSRT, *n* = 3; HSRT, *n* = 2), sedentary behavior (LSRT, *n* = 8; HSRT, *n* = 11), and exhaustion (LSRT, *n* = 5; HSRT, *n* = 5).

### Physical Function

The effects of RT on physical function in prefrail and frail are shown in Tables 2, 3 and Supplementary Figures 1, 2, respectively. LSRT and HSRT caused different patterns of improvements in physical function in prefrail. LSRT improved muscle strength of the right knee extensors (*P* = 0.01), right (*P* = 0.01) and left (*P* = 0.001) hip flexors, and right (*P* = 0.001) and left (*P* = 0.01) ankle extensors, while the right (*P* < 0.001) and left (*P* = 0.01) one-leg stand performances were significantly reduced. In contrast, TUG at fast pace (*P* = 0.01), TUG associated with a verbal task (*P* = 0.001), TUG associated with motor and verbal tasks (*P* < 0.001), and tandem balance (*P* = 0.01) were only improved after HSRT. Performance time (*P* < 0.001), power (*P* = 0.05, *P* < 0.001), and the velocity of muscle contraction (*P* < 0.001) in the sit-to-stand test, TUG at usual pace (*P* = 0.01, *P* < 0.001), and TUG associated with a motor task (*P* = 0.01, *P* < 0.001) were significantly improved in response to both LSRT and HSRT. CG showed a significant increase in the time on the sit-to-stand (*P* < 0.001) test. At the end of the protocol, higher TUG performance (*P* < 0.001) and muscle strength of the right (*P* < 0.001) and left knee extensors (*P* < 0.001) were observed in exercise groups in comparison to CG, while only LSRT showed lower right and left one-leg stand performances (*P* < 0.001) and higher muscle strength of the right (*P* = 0.01) and left (*P* < 0.01) hip flexors, and right (*P* < 0.01) and left (*P* < 0.01) ankle extensors in comparison to CG. Significant differences in TUG associated with motor task (*P* = 0.01), TUG associated with motor and verbal tasks (*P* = 0.01), and power (*P* = 0.01) in the sit-to-stand test were found between LSRT and HSRT.

**Table 2:** Effects of resistance training on physical performance of pre-frail older adults.

	**Baseline**			**16-week**		
	**LSRT**	**HSRT**	**CG**	**LSRT**	**HSRT**	**CG**
**Physical performance**						
Right IHG, kg	25.0 ± 4.0	21.9 ± 5.7	25.9 ± 3.2	27.4 ± 5.3	20.7 ± 4.0	25.7 ± 2.9
Left IHG, kg	25.5 ± 6.1	21.3 ± 6.0	25.7 ± 3.6	27.3 ± 6.3	20.0 ± 4.9	25.6 ± 3.0
Right knee extensor, kgf	17.3 ± 4.2	11.7 ± 2.3	10.1 ±1.9	19.2 ± 5.0^a^	13.5 ± 3.5	9.8 ± 1.8^b^^,^^c^
Left knee extensor, kgf	14.8 ± 3.1	12.3 ± 3.4	10.3 ± 2.3	16.8 ± 4.3	14.3 ± 3.8	9.7 ± 2.3
Right hip flexor, kgf	11.1 ± 3.2	8.2 ± 3.3	8.6 ± 3.6	12.8 ± 3.6^a^	9.1 ± 3.7	8.1 ± 3.3^b^
Left hip flexor, kgf	10.1 ± 2.7	8.1 ± 2.8	8.3 ± 2.5	12.5 ± 3.9^a^	8.7 ± 2.5	8.2 ± 2.6^b^
Right ankle extensor, kgf	6.8 ± 2.1	6.4 ± 1.8	5.8 ± 1.1	8.7 ± 2.9^a^	7.6 ± 1.5	5.6 ± 1.3^b^
Left ankle extensor, kgf	7.1 ± 1.7	6.4 ± 1.8	6.4 ± 1.1	8.7 ± 2.7^a^	7.5 ± 1.3	6.2 ± 1.3^b^
Right one-leg stand, s (30 s max)	19.4 ± 9.7	10.9 ± 11.6	12.5 ± 12.0	6.6 ± 0.7^a^	14.2 ± 12.0	11.9 ± 12.2^b^
Left one-leg stand, s (30 s max)	16.4 ± 11.0	13.0 ± 12.2	7.3 ± 10.4	5.5 ± 0.9^a^	17.9 ± 12.7	9.9 ± 11.5^b^
Normal balance, s (10 s max)	10.0 ± 0.0	9.8 ± 0.6	10.0 ± 0.0	10.0 ± 0.0	10.0 ± 0.0^a^	10.0 ± 0.0
Semi tandem balance, s (10 s max)	10.0 ± 0.0	9.8 ± 0.6	10.0 ± 0.0	10.0 ± 0.0	10.0 ± 0.0	10.0 ± 0.0
Tandem balance, s (10 s max)	10.0 ± 0.0	6.9 ± 0.6	10.0 ± 0.0	10.0 ± 0.0	7.3 ± 4.2^a^	10.0 ± 0.0
Sit-to-stand, s	8.4 ± 1.1	10.0 ± 2.3	8.0 ± 0.6	6.6 ± 0.8^a^	7.3 ± 2.0^a^	9.0 ± 1.1^a^
Sit-to-stand, power	49.0 ± 11.4	34.6 ± 8.8	46.5 ± 5.8	54.2 ± 13.3^a^	42.1 ± 12.6^a^^,^^b^	43.9 ± 5.6
Sit-to-stand, concentric contraction, m/s	1.3 ± 0.2	0.8 ± 0.3	1.2 ± 0.2	1.7 ± 0.4^a^	1.2 ± 0.4^a^	1.1 ± 0.1
Sit-to-stand, eccentric contraction, m/s	1.1 ± 0.5	1.2 ± 0.4	1.3 ± 0.4	1.0 ± 0.4	1.0 ± 0.4	1.3 ± 0.4
TUG at usual pace, s	8.0 ± 0.8	10.2 ± 2.7	6.2 ± 1.4	7.5 ± 0.8^a^	9.4 ± 2.5^a^	6.3 ± 1.4^b^^,^^c^
TUG at fast pace, s	6.5 ± 1.1	8.4 ± 2.5	5.6 ± 0.9	6.1 ± 1.0	7.8 ± 2.1^a^	6.0 ± 1.1
TUG with verbal task, s	8.3 ± 1.0	10.7 ± 3.9	7.1 ± 1.2	7.4 ± 0.5	9.1 ± 2.6^a^	7.4 ± 1.2
TUG with motor task, s	8.7 ± 1.7	10.1 ± 2.1	8.0 ± 0.8	7.8 ± 1.2^a^	9.4 ± 2.2^a^^,^^b^	8.2 ± 0.9
TUG with both verbal and motor tasks, s	8.3 ± 1.1	11.6 ± 3.2	10.9 ± 1.4	7.7 ± 0.8^a^	9.8 ± 2.5^a^^,^^b^	11.1 ± 1.2^a^
WS at usual pace, m/s	1.3 ± 0.3	1.2 ± 0.2	1.3 ± 0.3	1.5 ± 0.1	1.4 ± 0.2	1.2 ± 0.2
WS at fast pace, m/s	1.8 ± 0.3	1.5 ± 0.3	1.9 ± 0.3	1.8 ± 0.3	1.4 ± 0.2	2.1 ± 0.4
6MWT, m	480 ± 137	460 ± 151	589 ± 179	511 ± 135	478 ± 159	589 ± 179

*LSRT, Low-speed resistance training; HSRT, High-speed resistance training; CG, Control group. 6MWT, 6-min walking test; IHG, Isometric handgrip strength; TUG, Timed “Up and Go”; WS, Walking speed*;

a *P < 0.05 vs. Pre-intervention*;

b *P < 0.05 vs. LSRT*;

c *P < 0.05 vs. HSRT*.

**Table 3:** Effects of resistance training on physical performance of frail older adults.

	**Baseline**			**16-weeks**		
	**LSRT**	**HSRT**	**CG**	**LSRT**	**HSRT**	**CG**
**Physical performance**						
Right IHG, kg	6.2 ± 5.5	4.8 ± 6.4	13.8 ± 13.7	9.0 ± 9.9	7.0 ± 6.9	13.6 ± 13.9
Left IHG, kg	8.5 ± 9.5	9.6 ± 9.3	12.7 ± 12.4	11.2 ± 9.0	12.3 ± 12.0	13.7 ± 12.5
Right knee extensor, kgf	7.0 ± 1.9	7.1 ± 2.8	7.0 ± 5.7	10.6 ± 5.3	7.9 ± 7.2	6.8 ± 5.2
Left knee extensor, kgf	6.6 ± 2.0	6.1 ± 3.7	6.6 ± 5.0	9.7 ± 4.9^a^	9.2 ± 7.5^a^	6.2 ± 5.3
Right hip flexor, kgf	6.0 ± 1.7	5.4 ± 2.2	4.7 ± 2.8	7.4 ± 2.9^a^	6.6 ± 5.6^a^	5.0 ± 3.0
Left hip flexor, kgf	5.4 ± 1.1	5.1 ± 2.5	4.3 ± 2.5	6.8 ± 2.5^a^	7.1 ± 3.8^a^	4.7 ± 2.8
Right ankle extensor, kgf	5.6 ± 1.5	4.3 ± 2.6	3.8 ± 2.3	6.1 ± 1.7	4.3 ± 4.0	3.8 ± 2.6
Left ankle extensor, kgf	3.8 ± 2.8	4.4 ± 2.4	3.7 ± 2.6	4.5 ± 3.2	5.9 ± 3.0^a^	3.2 ± 2.3
Right one-leg stand, s (30 s max)	0.1 ± 0.3	0.1 ± 0.4	2.2 ± 3.1	1.0 ± 1.8	2.0 ± 5.7	2.8 ± 4.9
Left one-leg stand, s (30 s max)	0.0 ± 0.2	0.2 ± 0.4	2.3 ± 4.4	0.2 ± 0.4	1.7 ± 5.0	3.3 ± 7.8
Normal balance, s (10 s max)	1.2 ± 3.5	1.8 ± 4.0	4.4 ± 5.2	2.5 ± 4.6	2.7 ± 4.6	4.4 ± 5.2
Semi tandem balance, s (10 s max)	0.0 ± 0.0	1.0 ± 3.0	4.4 ± 5.2	1.2 ± 3.5	1.8 ± 4.0	4.4 ± 5.2
Tandem balance, s (10 s max)	0.0 ± 0.0	0.8 ± 2.7	5.5 ± 5.2	1.2 ± 3.5	0.9 ± 3.0	1.1 ± 3.3
Sit-to-stand, s	26.7 ± 11.6	26.2 ± 13.3	28.6 ± 10.9	17.1 ± 11.7^a^	18.9 ± 10.0	37.1 ± 19.3^b^^,^^c^
Sit-to-stand, power	13.9 ± 2.7	16.4 ± 7.1	11.9 ± 4.8	28.5 ± 10.6^a^	27.5 ± 13.8^a^	12.8 ± 3.6^b^^,^^c^
Sit-to-stand, concentric contraction, m/s	0.35 ± 0.30	0.26 ± 0.20	0.17 ± 0.10	0.55 ± 0.50	0.50 ± 0.43^a^	0.19 ± 0.0
Sit-to-stand, eccentric contraction, m/s	0.53 ± 0.51	0.53 ± 0.46	0.82 ± 0.46	0.65 ± 0.53	0.56 ± 0.46	0.86 ± 0.36
TUG at usual pace, s	119.8 ± 180.2	20.8 ± 27.3	46.4 ± 36.3	64.2 ± 4.7.4	23.9 ± 20.1	48.7 ± 37.4
TUG at fast pace, s	38.0 ± 46.3	17.4 ± 22.8	28.5 ± 25.4	45.0 ± 26.8	16.7 ± 16.7	25.8 ± 23.7
TUG with verbal task, s	69.0 ± 109.8	18.4 ± 24.1	37.5 ± 43.2	52.0 ± 50.0	20.8 ± 25.9	38.3 ± 45.3
TUG with motor task, s	14.2 ± 13.0	7.1 ± 12.9	16.1 ± 20.7	22.2 ± 22.2^a^	5.9 ± 11.1	13.7 ± 18.5
TUG with both verbal and motor tasks, s	17.6 ± 19.7	8.3 ± 18.7	17.7 ± 23.2	29.6 ± 39.0^a^	6.0 ± 13.8	17.1 ± 23.4
WS at usual pace, m/s	0.41 ± 0.37	0.81 ± 0.99	0.51 ± 0.41	0.41 ± 0.38	0.48 ± 0.38	0.58 ± 0.41
WS at fast pace, m/s	0.46 ± 0.41	0.66 ± 0.91	0.62 ± 0.50	0.48 ± 0.42	0.61 ± 0.34	0.65 ± 0.42
6MWT, m	150 ± 174	100 ± 136	91.4 ± 107			

*LSRT, Low-speed resistance training; HSRT, High-speed resistance training; CG, Control group. 6MWT, 6-min walking test; IHG, Isometric handgrip strength; TUG, Timed “Up and Go”; WS, Walking speed*;

a *P < 0.05 vs. Pre-intervention*;

b *P < 0.05 vs. LSRT*;

c *P < 0.05 vs. HSRT*.

RT improved fewer physical parameters in frail in comparison to prefrail. Power (*P* < 0.01) in the sit-to-stand test, muscle strength of the left knee extensors (*P* = 0.01) and right (*P* = 0.001) left (*P* = 0.001) hip flexors were improved after both LSRT and HSRT. Particularly, exclusive improvements in TUG associated with a motor task (*P* = 0.01), TUG associated with motor and verbal tasks (*P* = 0.01), and time in the sit-to-stand test (*P* = 0.01) were found in LSRT, while only HSRT improved muscle strength of the left ankle extensors (*P* = 0.001) and the velocity of the muscle concentric contraction in the sit-to-stand test (*P* = 0.01). Exercise groups showed higher performance (*P* = 0.001) and power (*P* = 0.001) in the sit-to-stand tests in comparison to CG. There were no significant differences among exercise groups.

Fourteen participants, six in the HSRT, four in the LSRT, and four in the CG, performed the sit-to-stand test with mobility aids or researchers' help at baseline. In contrast, four participants in the LSRT and three in the HSRT no longer needed help after exercise protocols.

### Cognitive Parameters

**Figure 3:**
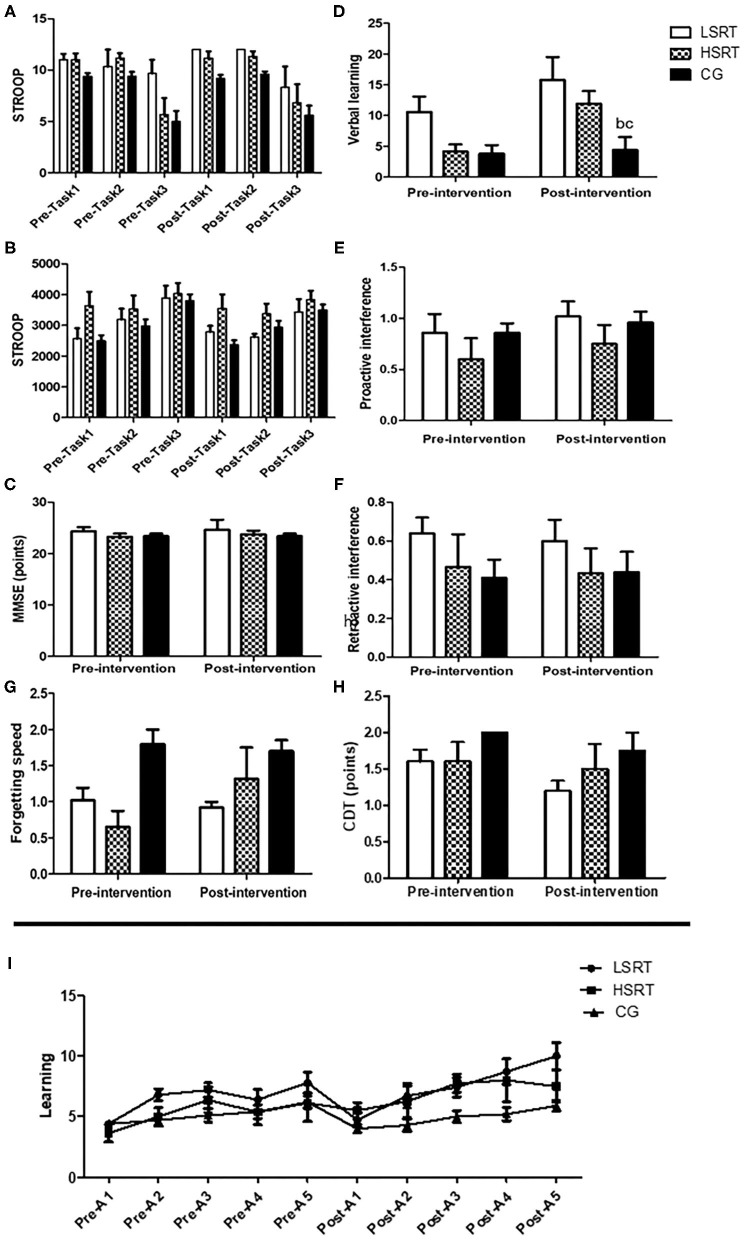
Effects of RT on cognitive parameters in prefrail older adults. Stroop test **(A,B)**, Mini-mental state examination (MMSE; **C**), The Rey's auditory verbal learning test **(D–G,I)** and Clock Drawing Tests **(H)**. LSRT, Low-speed resistance training; HSRT, High-speed resistance training; CG, Control group; MMSE, ^b^*P* < 0.05 vs. LSRT; ^c^*P* < 0.05 vs. HSRT.

**Figure 4:**
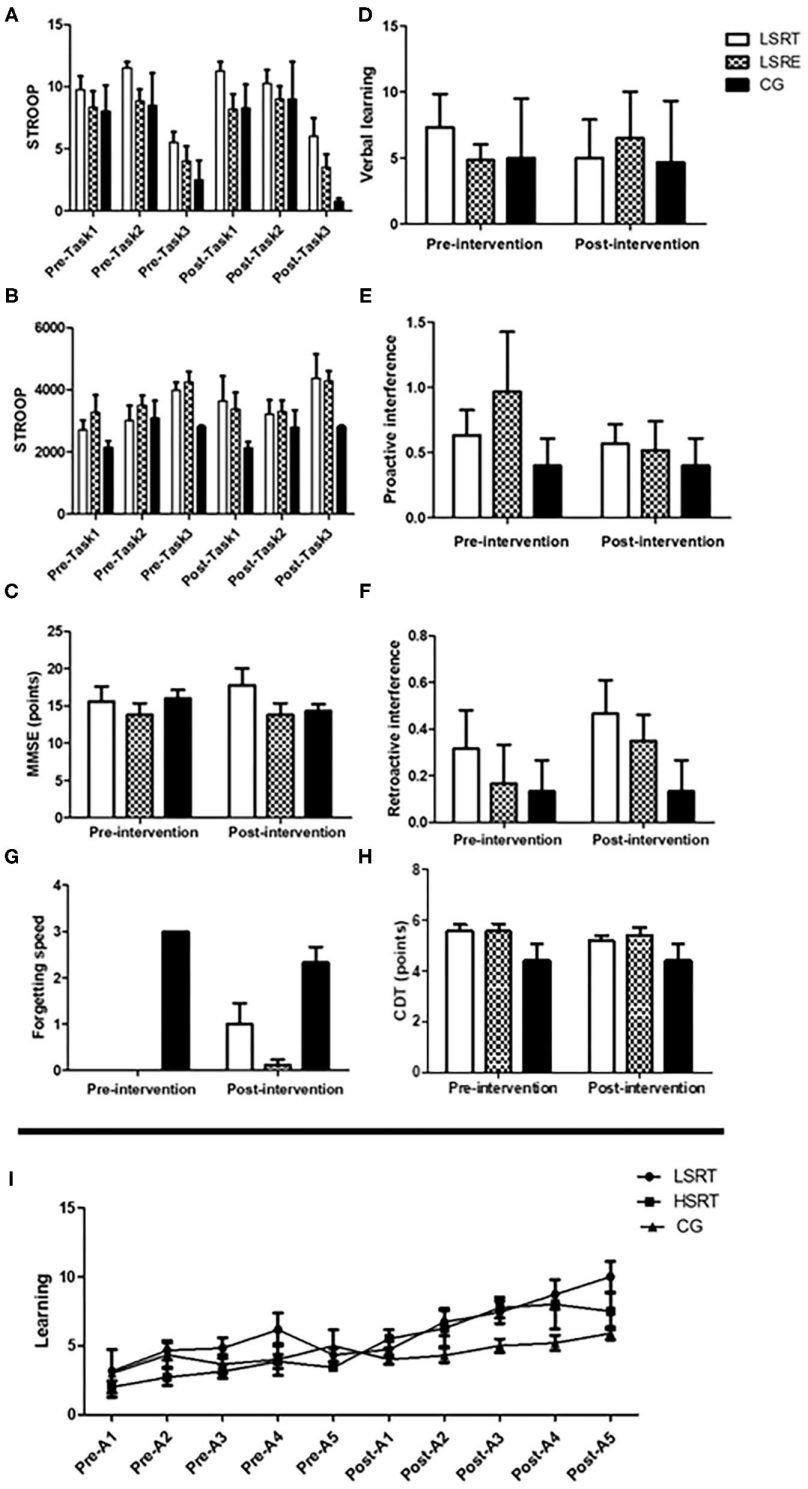
Effects of RT on cognitive parameters in prefrail older adults. Stroop test **(A,B)**, Mini-mental state examination (MMSE; **C**), The Rey's auditory verbal learning test **(D–G,I)** and Clock Drawing Tests **(H)**. LSRT, Low-speed resistance training; HSRT, High-speed resistance training; CG, Control group; MMSE.

The effects of RT on cognitive parameters in prefrail and frail people are shown in Figures 3, 4. There were no within- and between-group differences on MEEM, CDT, and STROOP in prefrail. On the other hand, higher verbal learning was observed after both LSRT and HSRT when compared to CG. In frail, no significant within- and between-group differences were observed on MEEM and STROOP performances. However, RAVLT performance (*P* = 0.01) was significantly improved after HSRT.

### Blood Pressure and Heart Rate

There were no within- and between-group differences on BP and HR in response to any intervention in prefrail and frail.

## Discussion

The main findings of the present study indicated that RT reversed frailty status and improved physical function in prefrail and frail older adults. Nevertheless, different improvements were observed among the groups in response to LSRT and HSRT. In addition, prefrail older adults showed higher RAVLT performance after both RT protocols in comparison to CG. Finally, no changes in BP and HR were observed in any group. A summary of the results is shown in Table 4.

**Table 4:** Effects of RT on frailty status, physical performance, cognitive function, and blood pressure and heart rate of prefrail and frail people.

	**Prefrail**			**Frail**		
**Variable**	**LSRT**	**HSRT**	**CG**	**LSRT**	**HSRT**	**CG**
**Frailty status**						
Weakness	↑	↔	↔	↑	↔	↔
Slow walking speed	↑	↑	↔	↔	↔	↔
Unintentional weight loss	↔	↔	↔	↑	↑	↔
Exhaustion	↑	↑	↔	↑	↑	↔
Low activity level	↔	↔	↔	↑	↑	↔
**Physical performance**						
Upper-limb muscle strength	↔	↔	↔	↔	↔	↔
Lower-limb muscle strength	↑↑	↑	↔	↑↑	↑	↔
Lower-limb muscle power	↑↑	↑	↔	↑	↑	↔
Mobility	↑	↑↑	↔	↔	↔	↔
Dual-task	↑	↑↑	↔	↑	↑	↔
Balance	**↓**	↑	↔	↔	↔	↔
**Cognitive function**						
Global	↔	↔	↔	↔	↔	↔
RAVLT	↑	↑	↔	↔	↔	↔
STROOP	↔	↔	↔	↔	↔	↔
**Hemodynamic parameters**						
SBP	↔	↔	↔	↔	↔	↔
DBP	↔	↔	↔	↔	↔	↔
HR	↔	↔	↔	↔	↔	↔

*LSRT, Low-speed resistance training; HSRT, High-speed resistance training; CG, Control group; SBP, Systolic blood pressure; DBP, Diastolic blood pressure; HR, Heart rate; ↑Improved vs. Pre-intervention; ↑↑Improved vs. Pre-intervention and CG and/or experimental group; ↓Reduced vs. Pre-intervetion; ↔Unchanged*.

### Effects of RT on Frailty Status

RT reversed frailty status in both prefrail and frail older adults. Our findings are supported by prior investigations that observed reductions in frailty status after exercise training protocols (77–83). However, most studies combined RT with other types of exercise and/or health interventions (81), limiting inferences regarding the impact of RT alone on frailty (84). In addition, the majority of the studies have focused on frailty components, whereas frailty status was only investigated in a few trials (33, 77).

Notably, RT improved weakness, slowness, and exhaustion in prefrail; and weight loss, sedentary behavior, and exhaustion in frail. Although surprising, similar results were found in the LIFE-P study (78), given that changes on frailty status were not associated with improvements on slowness and weakness, but physical activity levels.

A possible explanation for these findings is that prefrail individuals have more preserved physical function in comparison to frail counterparts, so that improvements on weakness (IHG) and slowness (WS) are easier to achieve cutoff values for robustness. In contrast, some frail participants in the present study had IHG values close to zero and took more than 60 s to perform WS test.

In this context, improvements in physical function may have contributed to reduce perceived fatigue (85), motivating frail participants to increase physical activity levels. Regarding weight loss, muscle hypertrophy is a well-established product of RT (86, 87) and it is possible to suggest that our RT programs reduced weight loss by modulating muscle mass.

These findings have important clinical implications by demonstrating that 16-weeks lower-limb LSRT and HSRT programs reversed frailty status in prefrail and frail older adults, possibly reducing the risk of negative events in these people (7–11). Particularly, some studies have reported low adherence to multicomponent exercise training programs, mainly in institutionalized frail older adults (80, 82), which might occur due to the fact the frail patients cannot support very-long exercise sessions (88). In addition, aerobic and gait exercises are not feasible and hard to prescribe in frail nursing home residents due to the high prevalence of mobility limitations (89). On the other hand, RT programs may be fully performed with individuals sitting in bed or in a chair without the need for transferring or walking, prioritizing some muscle groups, using body weight, free weights, or elastic bands (28, 90).

### Effects of RT on Muscle Strength and Power

Lower-limb muscle strength (i.e., knee extensors, hip flexors, ankle extensors) and power (i.e., time and power in the sit-to-stand) were significantly increased in prefrail and frail. Nevertheless, greater improvements were observed in LSRT relative to HSRT and CG.

These findings are in concordance with prior original articles (31, 79, 91–93) and systematic reviews (33) that investigated LSRT (79, 91, 92) and HSRT (91–93). However, just a few studies compared the effects of LSRT and HSRT in prefrail and, for the best of our knowledge, there are no investigations in frail people.

Several mechanisms may potentially explain why greater improvements were found after LSRT, including the time under tension (TUT), range of motion (ROM), the prevalence of comorbidities, and cognitive status.

Prior studies reported that TUT might impact strength gains in response to RT in healthy older adults (86, 94). Indeed, larger increases in dynamic and isometric strength have been observed in RT programs based on muscular contractions that lasted 6–7 s in comparison to those performed for ~2 s (86, 95, 96). Slow muscle contractions might reduce oxygen supply to the muscle (94) and increase the accumulation of products of cellular metabolism (95, 96). This scenario predisposes the recruitment of type II muscle fibers, those more associated with force generation and muscle hypertrophy (97), and additional motor units, according to the size principle of Henneman et al. (98), in an attempt to maintain force production (99). Hence, longer muscle contractions performed during LSRT (~5 s vs. ~2.5 s in HSRT) might have produced greater improvements on muscle strength by creating a more challenging metabolic environment, inducing the recruitment of type II muscle fibers and large motor units, resulting in superior neuromuscular adaptations.

Alternatively, the time under tension has been associated with increased myofibrillar protein synthesis and phosphorylation of anabolic signaling proteins (i.e., p70S6K, 4EBP1, and p90RSK) (100), likely inducing muscle hypertrophy (101). However, skeletal muscle mass was not assessed in the present study.

Notably, such greater improvements in muscle strength might have contributed to the development of muscle power in LSRT, given that force plays a key role in power production (102, 103) and muscle strength serve as the main driver for the ability to express high power outputs (103).

Another possible explanation for our results is based on the fact that most frail participants had reduced joint ROM due to high prevalence of lower limb osteoarthritis and the long-time using wheelchairs and mobility aids. The length-tension curve relationship states that exercises performed at optimal muscle length evokes greater myosin and actin interaction, and so strength (104), while exercises performed at partial ROM commonly produce less neuromuscular adaptations, restricted to the specific ROM in which muscle contractions occurred (105). Considering that sit-to-stand performance involves total knee and hip extensions, older adults with joint limitations might have performed exercises with reduced ROM, limiting the development of muscle strength and mainly power.

According to experts in the field (44), the prescription of HSRT to older adults with disabilities should take into consideration other factors than the variables of RT. Particularly, researchers have emphasized that participants must be continuously monitored and stimulated to keep concentric muscle contractions at high velocity (44). In the present study, exercise sessions were closely monitored and the HSRT protocol was composed by a few repetitions in an attempt to maintain participants' concentration. In addition, only older adults cognitively able to understand exercise and testing instructions were included. Nevertheless, the possibility that HSRT was not performed with the maximal power output cannot be ruled out.

### Effects of RT on Mobility, Dual-Task Performance, and Balance

HSRT is expected to produce greater improvement in mobility than LSRT (44, 45, 47, 90, 106). Bean et al. (39) found similar improvements in SPPB after non-equalized 16-weeks LSRT and HSRT programs in older adults. However, HSRT exhibited better effects when only older adults with mobility limitations were analyzed (39). Miszko et al. (37), Botaro et al. (107), and Ramírez-Campillo et al. (40) confirmed these findings by indicating that HSRT programs produced greater improvements in physical performance relative to LSRT, while Lopes et al. (41) reported exclusive improvements in sit-to-stand and TUG performances after HSRT.

Although these findings are supported by systematic review and metanalyses (42, 43), a wide confidence interval was observed between studies, suggesting that the effects of both LSRT and HSRT are still compatible with a clinically non-relevant difference. In addition, most studies were based on physically healthy older adults, short-term RT protocols, and expensive exercise machines, limiting extrapolations for prefrail and frail older adults.

In this context, findings of the present study are unique and add to the current knowledge by indicating that HSRT produced greater improvements in TUG performance in comparison to LSRT in prefrail older adults. A question that remains from these findings, then, is “how HSRT caused greater improvements in mobility without provoke larger increases in muscle strength and power?”

A likely explanation is that muscle power was improved in other muscle actions than those assessed in the present study. TUG involves the interaction among several body movements, including sit-to-stand transition, walking, turn and stand-to-sit transition (108). In fact, TUG performance requires power of the ankle flexors and extensors to stride velocity (35) and fast response to perturbations to turn (109).

Despite the similar improvements in muscle power, mobility was unaffected by LSRT and HSRT in frail. These results should be interpreted cautiously, given that most participants of the current study needed researchers' help or were not able to perform mobility tests at baseline, causing a wide variability in the results. Indeed, although no significant within-group differences were observed in WS and TUG, seven participants became independent to perform mobility tests after RT protocols. This scenario might also have influenced frailty status and indicates that long-term RT protocols seem to be necessary to reverse physical dysfunction in institutionalized frail older adults.

Notably, the improvements observed in muscle power might also account for the observed differences in balance in prefrail (36). However, it should be noted that all participants in LSRT and CG groups achieved the highest performance in normal and tandem tests in both pre- and post-intervention periods. In HSRT, only one participant did not complete the test at baseline but showed significant improvements after 16 weeks. These results suggest that LSRT and HSRT have limited effects on balance. In fact, neither LSRT nor HSRE significantly improved one-leg stand.

Another important finding is that prefrail participants showed better dual-task performance after HSRT, while LSRT was most effective in frail people. These results suggest that the effects of RT on dual-task performance might be dependent on frail status.

### Effects of RT on Cognitive Function, Blood Pressure, and Heart Rate

There is still no consensus on the effects of RT on the cognitive function of older adults (110) and only a few studies have examined prefrail and frail people. Mollinedo Cardalda et al. (93) and Yoon et al. (111) observed that RT improved overall cognitive function in frail older adults. This view was expanded by van de Rest et al. (52), who found increased digit span, attention, and working memory performances in prefrail and frail older adults who took part of a 24-weeks LSRT program. To the best of our knowledge, only Yoon et al. (112) compared the effects of HSRT and LSRT, and results revealed similar improvements in overall cognitive function.

The current study contributes to the growing literature by indicating that LSRT and HSRT improved verbal memory in community-dwelling prefrail older adults, regardless of the velocity of muscle contraction. However, our findings differ from prior investigations, given that no significant changes were found in global cognition, middle-term memory, inhibitory capacity, and attention in prefrail and frail older adults.

Differences in the results might be partially attributed to sample characteristics (52, 111, 112), since some studies combined prefrail and frail participants, cognitive status (e.g., mild-cognitive impairment) (93, 111–113), mobility levels (mobility-limited vs. able to walk) (93, 111, 112), cognitive assessment tools (52, 93, 111, 114), and RT programs (52, 93, 111, 114).

Our findings refuted the hypothesis that RT might reduce blood pressure and heart rate in prefrail and frail older adults. The majority of the studies on the effects of RT on blood pressure have examined robust community-dwelling older adults (112, 115–118) and no prior investigations included prefrail or frail participants. A possible explanation for our results may be the fact that the pathophysiology and progression of frailty involve the dysregulation of numerous mechanisms that predispose to increased blood pressure values (7, 119–121), which may not be counterregulated by neither LSRT nor HSRT.

### Practical Applications

Two main features of the current RT protocols should be highlighted. First, both LSRT and HSRT were low price, given that all equipment cost around $127,82, and seems feasible to public health programs. Second, the short duration of exercise sessions, which lasted ~25 min. Another practical aspect of the current study is that the reversion of frailty was influenced by the nursing home environment. Indeed, when frail participants showed minimal ability and resistance to walking few steps, a non-structured walking program was created. In this program, frail participants walked from 10 to 25 min at short intervals with the assistance of nursing students. It is worth mentioning that an affinity loop was created between researchers and study participants, and we deeply believe that this scenario contributed with participants' well-being and the adherence to exercise protocols. Finally, the question that remains is “What is the best RT protocol to improve frailty status and its related parameters in prefrail and frail older adults?” Taking into consideration all limitations of the present study, both exercise programs seem to be important in these populations improving different domains and reversing frailty status. Notably, LSRT seemed to be more effective in reverse prefrailty and frailty when compared to HSRT. Moreover, health practitioners should keep in mind that people with joint limitations and with probable cognitive impairments, as older adults living in LTI, might need more attention and auxiliary treatments (e.g., flexibility exercise) to properly perform HSRT. In any case, the next step would be to verify the effects of combined LSRT and HSRT programs.

### Limitations

Differences on age and on the context where participants were recruited are the two major limitations that avoid comparisons between pre-frail and frail older adults. Indeed, a mean difference of 10 years of age was observed between the groups. Age might indirectly influence the effects of RT on frailty and its associated parameters by impacting sedentary behavior, dietary habits, educational level, and social engagement (122–124). In addition, the main mechanisms underlying the effects of RT on neuromuscular function and cognition seems to be significantly affected by age (125–128). Regarding the setting of recruitment, older adults admitted to LTI are often socially isolated, have more depressive symptoms, a high prevalence of disability and multimorbidity, and increased cognitive decline (129–131). In the course of time, institutionalization can make things worse by contributing with the exacerbating of pre-existing conditions and with the development of new ones (132–135). Hence, it is possible that different results might be found in pre-frail and frail community-dwellers. However, it is important to note that the prevalence of frailty increases with age, and it is most commonly observed in LTI, with might explain our sample characteristics, so that future studies are still necessary to confirm our findings. Several additional limitations must be mentioned. First, participants were not screened for dementia since they were only required to understanding exercise commands. Second, the current findings are prevalently based on older women and extrapolations should be carefully performed. Third, although LSRT and HSRT had no effects on blood pressure, prior studies have noted that frailty was associated with ambulatory blood pressure, but not office blood pressure (18). Fourth, according to Vellas et al. (136) intervention periods longer than 12 months might be required to observe improvements in the cognitive function of older adults. Fifth, our sample size and inclusion criteria limited further analysis (e.g., respondents and non-respondents) (137, 138). Sixth, the possible mechanisms underlying the effects of RT on physical function were not investigated. Seventh, prefrail and frail older adults were recruited from different settings. Eighth, sample size calculation was based on changes on muscle strength, so that it might not be adequate to the other study outcomes, including frailty. Finally, additional covariables [e.g., high inflammatory status (139)] that could influence the current results were not controlled.

### Conclusions

Findings of the present study indicated that both LSRT and HSRT reversed frailty status and improved physical performance in prefrail and frail older adults. Notably, different patterns of improvement were observed among RT protocols. Regarding frailty status, LSRT seemed to be more effective in reverse prefrailty and frailty when compared to HSRT. Greater improvements in muscle strength and power were also observed after LSRT, while HSRT produced superior increases in mobility and dual-task performance. One-leg stand performance was significantly reduced in LSRT, but not HSRT and CG, after 16 weeks. In contrast, RT programs similarly improved verbal memory in prefrail. Finally, no changes in BP and HR were observed, regardless of the type of RT.

## Data Availability Statement

The raw data supporting the conclusions of this article will be made available by the authors, without undue reservation.

## Ethics Statement

The studies involving human participants were reviewed and approved by University of Campinas Human Research Ethics Committee. The patients/participants provided their written informed consent to participate in this study.

## Author Contributions

HC-J and MU: methodology, analysis, writing-original draft preparation, and writing-review and editing. HC-J: data collection and project administration. MU: supervision. Both authors contributed to the article and approved the submitted version.

## Conflict of Interest

The authors declare that the research was conducted in the absence of any commercial or financial relationships that could be construed as a potential conflict of interest.

## Publisher's Note

All claims expressed in this article are solely those of the authors and do not necessarily represent those of their affiliated organizations, or those of the publisher, the editors and the reviewers. Any product that may be evaluated in this article, or claim that may be made by its manufacturer, is not guaranteed or endorsed by the publisher.

## Supplementary Material

Supplementary Figure 1 Effects of RT on physical performance in prefrail older adults. LSRT, Low-speed resistance training; HSRT, High-speed resistance training; CG, Control group. 6MWT, 6-minute walking test; IHG, Isometric handgrip strength; TUG, Timed “Up and Go”; WS, Walking speed; ^a^*P* < 0.05 vs. Pre-intervention; ^b^*P* < 0.05 vs. LSRT; ^c^*P* < 0.05 vs. HSRT.

Supplementary Figure 2 Effects of RT on physical performance in frail older adults. LSRT, Low-speed resistance training; HSRT, High-speed resistance training; CG, Control group. 6MWT, 6-minute walking test; IHG, Isometric handgrip strength; TUG, Timed “Up and Go”; WS, Walking speed; ^a^*P* < 0.05 vs. Pre-intervention; ^b^*P* < 0.05 vs. LSRT; ^c^*P* < 0.05 vs. HSRT.
