# Supplementary figures and images for: Effects of Low-Speed and High-Speed Resistance Training Programs on Frailty Status, Physical Performance, Cognitive Function, and Blood Pressure in Prefrail and Frail Older Adults
Source: Front Med (Lausanne). 2021 Jul 26;8:702436. doi: 10.3389/fmed.2021.702436 (PMC8350041; doi:10.3389/fmed.2021.702436)

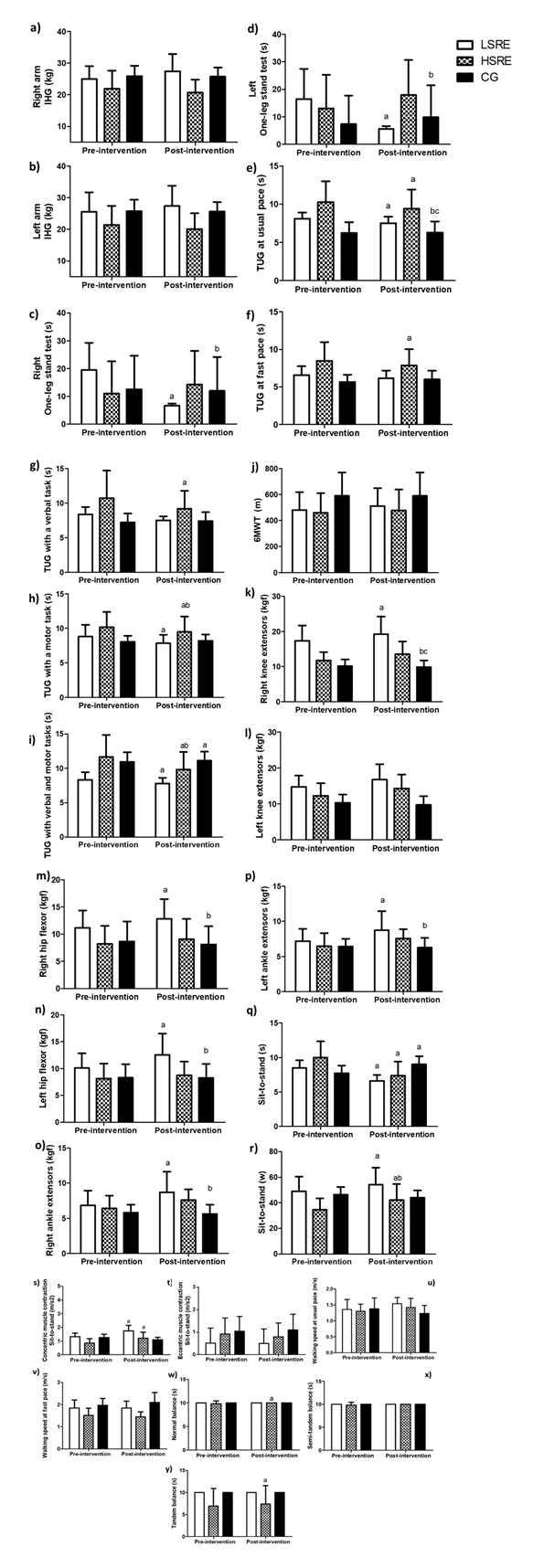

Supplement: Supplementary Figure 1 — Effects of RT on physical performance in prefrail older adults. LSRT, Low-speed resistance training; HSRT, High-speed resistance training; CG, Control group. 6MWT, 6-minute walking test; IHG, Isometric handgrip strength; TUG, Timed “Up and Go”; WS, Walking speed; aP < 0.05 vs. Pre-intervention; bP < 0.05 vs. LSRT; cP < 0.05 vs. HSRT. [file Image_1.TIF]

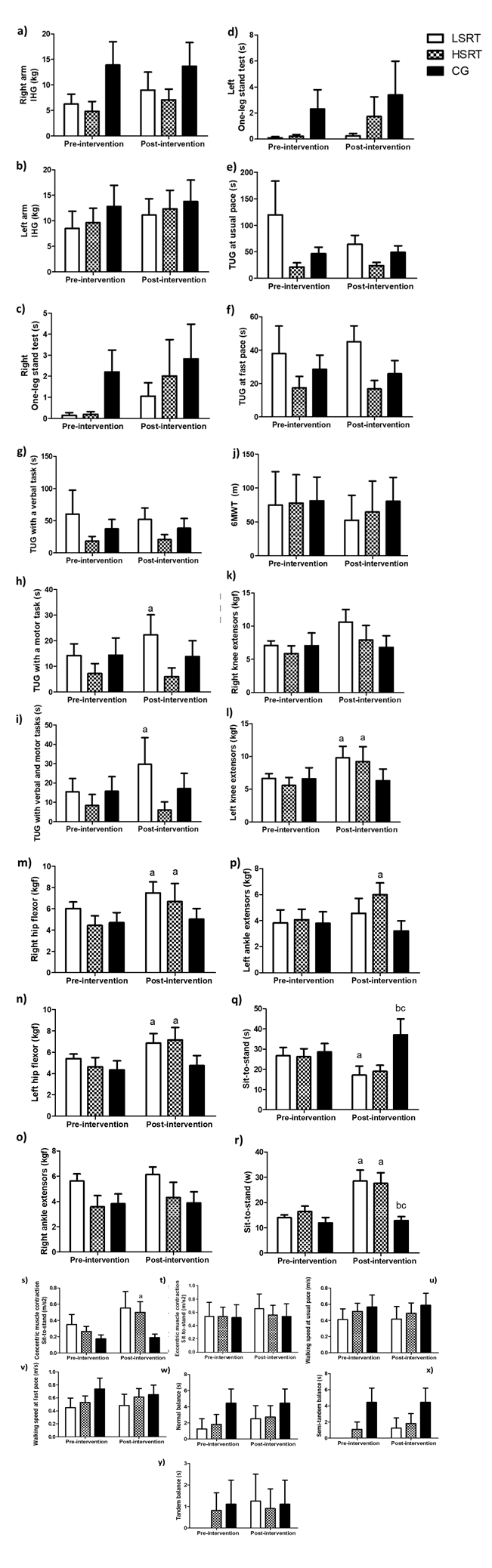

Supplement: Supplementary Figure 2 — Effects of RT on physical performance in frail older adults. LSRT, Low-speed resistance training; HSRT, High-speed resistance training; CG, Control group. 6MWT, 6-minute walking test; IHG, Isometric handgrip strength; TUG, Timed “Up and Go”; WS, Walking speed; aP < 0.05 vs. Pre-intervention; bP < 0.05 vs. LSRT; cP < 0.05 vs. HSRT. [file Image_2.TIF]
