# Supplementary material for: Effects of Low-Speed and High-Speed Resistance Training Programs on Frailty Status, Physical Performance, Cognitive Function, and Blood Pressure in Prefrail and Frail Older Adults
Source: Front Med (Lausanne). 2021 Jul 26;8:702436. doi: 10.3389/fmed.2021.702436 (PMC8350041; doi:10.3389/fmed.2021.702436)
Supplement: Supplementary file 3 [file Table_1.DOCX]

Supplementary Material

# Physical Performance Tests

*1.1 Isometric Handgrip Strength*

IHG of the dominant and nondominant hands was measured using a Jamar® handheld hydraulic dynamometer (Sammons Preston, Bolingbrook, IL, USA) [1]. The measure was obtained while the participant was seated on a chair with the shoulder abducted, the elbow near the trunk and flexed at 90°, and the wrist in a neutral position (thumbs up). The contralateral arm remained relaxed under the thigh. To measure IHG, participants performed a maximal contraction during 4 s. The test reliability in prefrail and frail participants was 0.97 and 0.98, respectively.

*1.2 Isometric muscle strength of knee extensors, hip flexors, and ankle extensors*

The isometric strength of knee extensors, hip flexors, and ankle extensors was measured using a handheld dynamometer (mTasF-1; ANIMA, Tokyo, Japão) [2]. Before testing, participants remained seated with both hands on their thighs, and their knee and hip flexed at 90°. Muscle strength was assessed with the handheld placed: a) near the midpoint of the tibia, for knee extensors; b) near the midpoint of the femur, for hip flexors; and c) between the patella and the femur, for ankle extensors. Participants were requested to perform as much strength was possible to move their joints for 4 seconds. The test reliability in prefrail and frail participants was 1.0.

*1.3 One-Leg Stand Test*

The one-leg stand test was performed with participants standing in a unipodal stance on the dominant and nondominant lower limbs, with the contralateral knee flexed at 90°, arms folded across the chest or stretched over the body, and head held straight [3]. Timing began when participants raised one foot off the floor and was stopped when the foot touched the floor again. The maximum performance time was set at 30 s. The test reliability in prefrail and frail participants was 0.8.

*1.4 Balance tests of the SPPB*

Participants performed the hierarchical test of standing balance of the SPPB [4]. Participants were asked to stand with their feet side by side, followed by the semitandem (heel of one foot alongside the big toe of the other foot) and tandem (heel of one foot directly in front of and touching the other foot) positions for 10 s each. The test reliability in prefrail and frail participants was 0.8.

*1.5 Sit-to-Stand Test*

Participants rose from a chair five times as quick as possible with at least one arm positioned on the waist, while the other arm remained folded across the chest or holding a researchers’ hand. Timing began when the participant raised their buttocks off the chair and was stopped when the participant was seated at the end of the fifth stand [4]. A 50-Hz linear encoder (Peak Power, CEFISE, Brazil) was attached to the wrist of the arm that was at the waist to obtain muscle power (w) and the velocity (m/s²) of concentric and eccentric contractions. The test reliability in prefrail and frail participants was 1.0 and 7.8, respectively.

*1.6 Timed-Up-and-Go Test*

The TUG test involved getting up from a chair (total height: 87 cm; seat height: 45cm; width: 33 cm), walking three meters around a cone placed on the floor, coming back to the same position, and sitting back on the chair [5]. Participants wore regular footwear, placed their back against the chair, rested their arms on the chair's arms, and put their feet on the ground. A researcher instructed the participant to, on the word “go”, get up, walk three meters as fast as possible without compromising the safety, turn, walk three meters back to the chair, and sit down. Timing began when the participant got up from the chair and was stopped when participant’s back touched the backrest of the chair. TUG was performed at usual and fast paces in the present study. The test reliability in prefrail and frail participants was 0.9.

*1.7 Timed-Up-and-Go Test with secondary tasks*

After performing the traditional TUG task, participants performed b) TUG combined with a verbal ﬂuency task, naming as many animals as they could remember, (c) TUG with a motor task, carrying a full cup of water), and (d) TUG test with both cognitive and motor tasks; i.e., performing the verbal ﬂuency test while carrying a full cup of water [6]. The test reliability in prefrail and frail participants was 0.98 and 0.94, respectively.

*1.8 Walking Speed Tests*

WS was measured over four meters[4]. For the test, participants were required to walk six meters (including one-meter acceleration and one-meter deceleration) at their usual and fastest possible pace (without running). Before the evaluation, both feet of each participant were to remain on the starting line. Timing began when a foot reached the 1-meter line and was stopped when a foot reached the 4-meter line. The 1-meter intervals at the beginning and at the end of the course were used to avoid early acceleration and/or deceleration. The test reliability in prefrail and frail participants was 1.0.

*1.9 6-min walking test*

The 6MWT was performed according to the American Thoracic Society guidelines [7]. The test was performed indoors on a 30-m track. In summary, after remaining seated for 15 min, the volunteers were asked to walk on the track as fast as possible for six minutes. In the case that the volunteers experienced chest pain, intolerable dyspnea, leg cramps, stagger, diaphoresis, pale or ashen appearance, or any other complaint, the test was interrupted. The distance walked by the volunteers in meters was used in the analysis.

**2. Cognitive Function**

*2.1 Mini-Mental State Examination (MMSE)*

The participants’ cognitive function was assessed using the MMSE, which is a standard test in cognitive aging research to assess mental status with a possible score of 0–30. MMSE evaluates orientation, registration, and short-term recall, attention and concentration, language (naming, sentence writing, and comprehension), and visuospatial abilities. Individual items are summed to generate the total score. If individuals decline or are unable to attempt a task, the value of that particular item would be missing (1,2).

*2.2 Clock Drawing Test (CDT)*

CDT involves draw the face of a large clock, place all the numbers inside the clock and place the pointers indicating 11:10 (eleven hours and ten minutes). No time limit was given to participants and they were allowed to draw as many watches as they wanted as long as only one of them was indicated for analysis. CDT was analyzed according to the method proposed by Shulman et al. (3).

*2.3 Rey's Auditory Verbal Learning Test (RAVLT)*

RAVLT is a neuropsychological tool used for testing episodic memory (4–7) and its scores have been strongly associated with the atrophy of medial temporal lobe structures (e.g., hippocampus) responsible for memory formation and maintenance after learning (6). In addition, RAVLT is useful to distinguish patients with and without dementia (4) and normative data according to gender and age have been provided to young, middle-aged and older adults (7,8) and patients with stroke, epilepsy, and neoplasm (5). The test consists of read-aloud two lists (A and B) of 15 substantives each (with a 1-s interval between each word). At the beginning of the test, list A was read five consecutive times by a researcher. Then, participants were requested to recall as many words were possible after each trial (A1-A5). The list B, interference list, with new 15 substantives was read after A5 and words were retrieved (B1). Finally, participants were asked to recall the words from list A immediately after the interference list (A6, immediate recall) and after a delay of 20 minutes (A7, delayed recall), without listening to the list A again (8).

*2.4 Stroop test*

A computerized version of the Stroop test (TESTINPACSTM) was used to provide reaction time (ms) and the number of correct words in each stimulus (control, congruent, incongruent) (9,10). To the test, participants remained seated in front of a 17-inch color monitor. The distance between the participant and the monitor was chosen according to the participants’ vision needs. Stroop was divided into three phases. In the first phase, control stimulus, the monitor exhibited a rectangle painted in green, yellow, blue, or red. Two possible responses, corresponding or not to the color of the rectangle, were exhibited at the lower corners of the monitor, and participants were requested to tell the color corresponding to the rectangle. The second phase was called congruent stimulus and consisted in stimulus (i.e., name of a color) and responses (i.e., name of two colors, one corresponding to the first color and the other not) exhibited as words in white. The correct answer was telling which colors match. The third phase, incongruent stimulus, is called Stroop effect and consisted of four colors exhibited is an incompatible color. The participants were requested to tell the color corresponding to the letters and inhibit the response for the identity of the disclosed word. A total of 36 stimuli (12 attempts each phase) were randomly provided and the time was registered in milliseconds. After the participants’ response, a researcher was responsible to immediately press the corresponding key (← or →). This protocol was established after a pilot study in which we observed that participants of the present study took too long or were not able to return the hand to the initial position, if they had to take it off, even if the keyboard was composed only by two keys.

**3. References**

1. Coelho-Júnior HJ, Gambassi BB, Irigoyen M-C, Gonçalves IDO, Oliveira PDLL, Schwingel PA, Alves CHL, Asano RY, Uchida MC, Rodrigues B. Hypertension, Sarcopenia, and Global Cognitive Function in Community-Dwelling Older Women: A Preliminary Study. *J Aging Res* (2018) **2018**: doi:10.1155/2018/9758040

2. Brucki SMD, Nitrini R, Caramelli P, Bertolucci PHF, Okamoto IH. Sugestões para o uso do mini-exame do estado mental no Brasil. *Arq Neuropsiquiatr* (2003) **61**:777–781. doi:10.1590/S0004-282X2003000500014

3. Shulman KI, Pushkar Gold D, Cohen CA, Zucchero CA. Clock‐drawing and dementia in the community: A longitudinal study. *Int J Geriatr Psychiatry* (1993) **8**:487–496. doi:10.1002/gps.930080606

4. Estévez-González A, Kulisevsky J, Boltes A, Otermín P, García-Sánchez C. Rey verbal learning test is a useful tool for differential diagnosis in the preclinical phase of Alzheimer’s disease: comparison with mild cognitive impairment and normal aging. *Int J Geriatr Psychiatry* (2003) **18**:1021–1028. doi:10.1002/gps.1010

5. Schoenberg MR, Dawson KA, Duff K, Patton D, Scott JG, Adams RL. Test performance and classification statistics for the Rey Auditory Verbal Learning Test in selected clinical samples. *Arch Clin Neuropsychol* (2006) **21**:693–703. doi:10.1016/J.ACN.2006.06.010

6. Moradi E, Hallikainen I, Hänninen T, Tohka J, Alzheimer’s Disease Neuroimaging Initiative ADN. Rey’s Auditory Verbal Learning Test scores can be predicted from whole brain MRI in Alzheimer’s disease. *NeuroImage Clin* (2017) **13**:415–427. doi:10.1016/j.nicl.2016.12.011

7. Magalhães SS, Hamdan AC. The Rey Auditory Verbal Learning Test: Normative data for the Brazilian population and analysis of the influence of demographic variables. *Psychol Neurosci* (2010) **3**:85–91. doi:10.3922/j.psns.2010.1.011

8. Malloy-Diniz LF, Lasmar VAP, Gazinelli L de SR, Fuentes D, Salgado JV. The Rey Auditory-Verbal Learning Test: applicability for the Brazilian elderly population. *Rev Bras Psiquiatr* (2007) **29**:324–329. doi:10.1590/S1516-44462006005000053

9. Barbosa DF, Prada FJA, Glanner MF, Nóbrega O de T, Córdova CO de A. [Cardiovascular response to Stroop test: comparison between the computerized and verbal tests]. *Arq Bras Cardiol* (2010) **94**:507–11. doi:10.1590/s0066-782x2010005000006

10. Franco-Alvarenga PE, Brietzke C, José Coelho-Júnior H, Canestri R, Nagata EY, Asano RY, Pires FO. Physical education class can improve acute inhibitory control in elementary school students. *Mot Rev Educ Física* (2019) **25**: doi:10.1590/s1980-6574201900020007
